# Supplementary material for: Semi-Synthesis of N-Aryl Amide Analogs of Piperine from Piper nigrum and Evaluation of Their Antitrypanosomal, Antimalarial, and Anti-SARS-CoV-2 Main Protease Activities
Source: Molecules. 2022 Apr 29;27(9):2841. doi: 10.3390/molecules27092841 (PMC9100884; doi:10.3390/molecules27092841)
Supplement: Supplementary file 1 [file molecules-27-02841-s001.zip › molecules-1656788-supplementary.pdf]

Supplementary Materials

Semi-Synthesis of *N*-Aryl Amide Analogs of Piperine from *Piper nigrum* and Evaluation of their Antitrypanosomal, Antimalarial, and Anti-SARS-CoV-2 Main Protease Activities

Rattanaporn Wansri [1,2,3], Aye Chan Khine Lin [1,2,3], Jutharat Pengon [4], Sumalee Kamchonwongpaisan [4], Nitipol Srimongkolpithak [4], Roonglawan Rattanajak [4], Patcharin Wilasluck [5,6], Peerapon Deetanya [5,6], Kittikhun Wangkanont [5,6], Kowit Hengphasatporn [7], Yasuteru Shigeta [7], Jatupol Liangsakul [8], Aphinya Suroengrit [9], Siwaporn Boonyasuppayakorn [9], Taksina Chuanasa [2], Wanchai De-eknamkul [2], Supot Hannongbua [9], Thanyada Rungrotmongkol [10,11], Supakarn Chamni [1,2,3,*]

1 Pharmaceutical Sciences and Technology Program, Faculty of Pharmaceutical Sciences, Chulalongkorn University, Bangkok 10330, Thailand
2 Department of Pharmacognosy and Pharmaceutical Botany, Faculty of Pharmaceutical Sciences, Chulalongkorn University, Bangkok 10330, Thailand
3 Natural Products and Nanoparticles Research Unit (NP²), Chulalongkorn University, Bangkok 10330, Thailand; supakarn.c@pharm.chula.ac.th
4 National Center for Genetic Engineering and Biotechnology (BIOTEC), National Science and Technology Development Agency, Pathum Thani 12120, Thailand; nitipol.sri@biotec.or.th
5 Center of Excellence for Molecular Biology and Genomics of Shrimp, Department of Biochemistry, Faculty of Science, Chulalongkorn University, Bangkok 10330, Thailand; Kittikhun.W@chula.ac.th
6 Molecular Crop Research Unit, Department of Biochemistry, Faculty of Science, Chulalongkorn University, Bangkok 10330, Thailand
7 Center for Computational Sciences, University of Tsukuba, 1-1-1 Tennodai, Tsukuba, Ibaraki 305-8577, Japan; shigeta@ccs.tsukuba.ac.jp
8 Scientific and Technological Research Equipment Centre Chulalongkorn University, Bangkok, 10330, Thailand; Jatu.P@chula.ac.th
9 Applied Medical Virology Research Unit, Department of Microbiology, Faculty of Medicine, Chulalongkorn University; siwaporn.b@chula.ac.th
10 Natural Product Biotechnology Research Unit, Chulalongkorn University, Bangkok 10330, Thailand; wanchai.d@chula.ac.th
11 Center of Excellence in Computational Chemistry (CECC), Department of Chemistry, Faculty of Science, Chulalongkorn University, Bangkok 10330, Thailand; supot.h@chula.ac.th
12 Center of Excellence in Biocatalyst and Sustainable Biotechnology, Department of Biochemistry, Faculty of Science, Chulalongkorn University, Bangkok 10330, Thailand; thanyada.r@chula.ac.th
13 Program in Bioinformatics and Computational Biology, Graduate School, Chulalongkorn University, Bangkok 10330, Thailand

* Correspondence: supakarn.c@pharm.chula.ac.th; Tel.: +662-218-8357

Content	Page
**Table S1** Druglikeness, predict ADME parameters, and pharmacokinetic properties	S3
**Figure S1** RMSD analysis of piperine (**1**) and 2,5 dimethoxy substituted phenyl piperamide **5**	S3
**Table S2** ^{1}H and ^{13}C chemical shifts (δ in ppm, *J* in Hz) of piperine (**1**) and piperic acid (**2**) in acetone-d_6 (400 mHz)	S4
**Table S3** ^{1}H and ^{13}C chemical shifts (δ in ppm, *J* in Hz) of *N*-aryl amide derivative of piperine **3**-**5** in acetone-d_6 (400 mHz)	S5
**Figure S2** ^{1}H–NMR spectrum of piperine (**1**) in acetone-d_6	S6
**Figure S3** ^{13}C–NMR spectrum of piperine (**1**) in acetone-d_6	S6
**Figure S4** COSY spectrum of piperine (**1**) in acetone-d_6	S7
**Figure S5** IR spectrum of piperine (**1**), ATR	S7
**Figure S6** ^{1}H–NMR spectrum of piperic acid (**2**) in acetone-d_6	S8
**Figure S7** ^{13}C–NMR spectrum of piperic acid (**2**) in acetone-d_6	S8
**Figure S8** COSY spectrum of piperic acid (**2**) in acetone-d_6	S9
**Figure S9** IR spectrum of piperic acid (**2**), ATR	S9
**Figure S10** ^{1}H–NMR spectrum of compound **3** in acetone-d_6	S10
**Figure S11** ^{13}C–NMR spectrum of compound **3** in acetone-d_6	S10
**Figure S12** COSY spectrum of compound **3** in acetone-d_6	S11
**Figure S13** HSQC spectrum of compound **3** in acetone-d_6	S11
**Figure S14** HMBC spectrum of compound **3** in acetone-d_6	S12
**Figure S15** IR spectrum of compound **3**, ATR	S12
**Figure S16** ^{1}H–NMR spectrum of compound **4** in acetone-d_6	S13
**Figure S17** ^{13}C–NMR spectrum of compound **4** in acetone-d_6	S13
**Figure S18** COSY spectrum of compound **4** in acetone-d_6	S14
**Figure S19** HSQC spectrum of compound **4** in acetone-d_6	S14
**Figure S20** HMBC spectrum of compound **4** in acetone-d_6	S15
**Figure S21** IR spectrum of compound **4**, ATR	S15
**Figure S22** ^{1}H–NMR spectrum of compound **5** in acetone-d_6	S16
**Figure S23** ^{13}C–NMR spectrum of compound **5** in acetone-d_6	S16
**Figure S24** COSY spectrum of compound **5** in acetone-d_6	S17
**Figure S25** HSQC spectrum of compound **5** in acetone-d_6	S17
**Figure S26** HMBC spectrum of compound **5** in acetone-d_6	S18
**Figure S27** IR spectrum of compound **5**, ATR	S18

Table S1. Drug-likeness, predict ADME parameters, and pharmacokinetic properties (www.swissadme.ch)

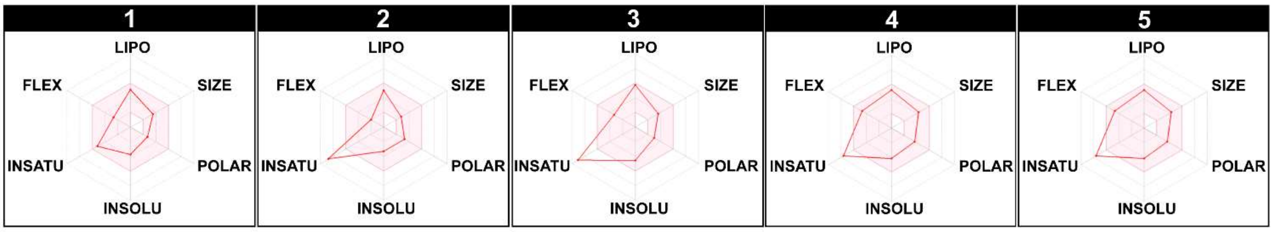

Properties	Compounds				
	Piperine (**1**)	Piperic acid (**2**)	3	4	5
Formula	$C_{17}H_{19}NO_3$	$C_{12}H_{10}O_4$	$C_{18}H_{15}NO_3$	$C_{20}H_{19}NO_5$	$C_{20}H_{19}NO_5$
Molecular weight	285.34 g/mol	218.21 g/mol	293.32 g/mol	353.37 g/mol	353.37 g/mol
H-bond acceptors	3	4	3	5	5
H-bond donors	0	1	1	1	1
Lipophilicity Log $P_{o/w}$ (iLOGP)	3.38	2.20	3.00	3.82	3.71
Water Solubility Log *S* (Ali)	-3.96<br>0.111 mmol/l	-4.07<br>0.084 mmol/l	-5.26<br>0.005 mmol/l	-4.65<br>0.008 mmol/l	-4.65<br>0.002 mmol/l
Bioavailability Score	0.55	0.85	0.55	0.55	0.55

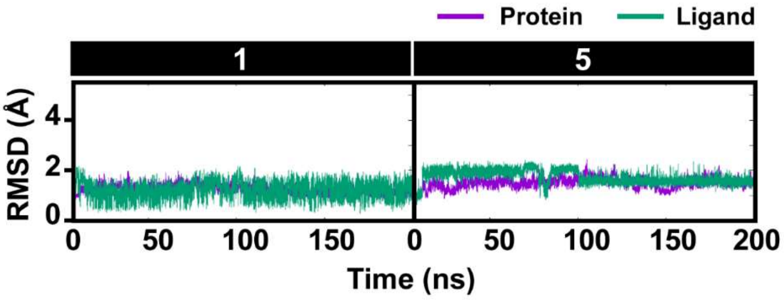

Figure S1. RMSD analysis of piperine (**1**) and 2,5 dimethoxy substituted phenyl piperamide **5**

Table S2. ^{1}H and ^{13}C chemical shifts (δ in ppm, *J* in Hz) of piperine (**1**) and piperic acid (**2**) in acetone, *d*$_6$ (400 mHz)

Carbon No.	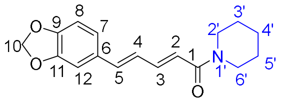 Piperine (**1**)		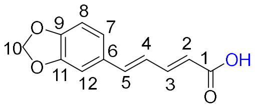 Piperic acid (**2**)	
	^{1}H NMR	^{13}C NMR	^{1}H NMR	^{13}C NMR
1	-	165.3	-	167.9
2	6.65 (d, *J* = 14.4 Hz, 1H)	121.9	6.00 (d, *J* = 15.2 Hz, 1H)	121.4
3	7.31 (dd, *J* = 10.4 and 14.4 Hz, 1H)	142.6	7.40 (m, 1H)	146.1
4	6.93 (d, *J* = 10.8 Hz, 1H)	126.7	6.98 (d, *J* = 10.0 Hz, 1H)	125.7
5	6.85 (d, *J* = 8.0 Hz, 1H)	138.9	6.97 (d, *J* = 5.6 Hz, 1H)	141.0
6	-	131.7	-	131.9
7	6.99 (dd, *J* = 1.6 and 8.0 Hz, 1H)	123.4	7.03 (dd, *J* = 1.6 and 8.0 Hz, 1H)	124.0
8	6.98 (d, *J* = 8.0 Hz, 1H)	109.3	6.86 (d, *J* = 8.0 Hz, 1H)	109.3
9	-	149.1	-	149.5
10	6.02 (s, 2H)	102.3	6.04 (s, 2H)	102.5
11	-	149.3	-	149.6
12	7.11 (d, *J* = 1.6 Hz, 1H)	106.4	7.17 (d, *J* = 1.6 Hz, 1H)	106.6
1′	-	-		-
2′	3.57 (t, *J* = 5.4 Hz, 2H)	46.4	-	-
3′	1.53 (m, 2H)	27.6	-	-
4′	1.64 (m, 2H)	25.5	-	-
5′	1.53 (m, 2H)	26.6	-	-
6′	3.57 (t, *J* = 5.4 Hz 2H)	43.5	-	-

Table S3. ^{1}H and ^{13}C chemical shifts (δ in ppm, *J* in Hz) of *N*-aryl amide derivative of piperine **3-5** in acetone, d_6 (400 mHz)

Carbon	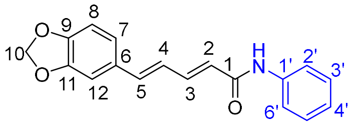 **3**		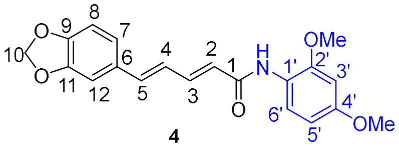 **4**		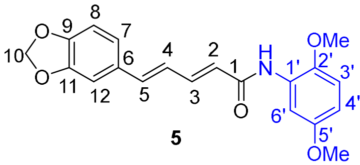 **5**	
	No.^{1}H NMR	^{13}C NMR	^{1}H NMR	^{13}C NMR	^{1}H NMR	^{13}C NMR
1	-	164.9	-	164.4	-	164.8
2	6.31 (d, *J* = 14.8 Hz, 1H)	125.3	6.46 (d, *J* = 14.8 Hz, 1H)	125.8	6.49 (d, *J* = 14.8 Hz, 1H)	125.5
3	7.43 (ddd, *J* = 3.6, 7.2, 15.2 Hz, 1H)	142.3	7.40 (ddd, *J* = 4.6, 6.0 and 14.8 Hz 1H)	141.7	7.42 (ddd, *J* = 2.4, 8.0, 14.8 Hz, 1H)	142.4
4	6.94 (d, *J* = 6.8 Hz, 1H)	126.0	6.93 (d, *J* = 6.0 Hz, 1H)	126.1	6.95 (d, *J* = 7.2 Hz, 1H)	139.9
5	6.93 (d, *J* = 3.6 Hz, 1H)	139.8	6.92 (d, *J* = 4.4 Hz, 1H)	139.4	6.94 (d, *J* = 8.8 Hz, 1H)	126.0
6	-	132.1	-	132.2	-	132.1
7	7.02 (dd, *J* = 1.6 and 8.0 Hz, 1H)	123.7	7.02 (dd, *J* = 1.6 and 8.0 Hz, 1H)	123.6	7.02 (dd, *J* = 1.4 and 8.2 Hz, 1H)	123.7
8	6.85 (d, *J* = 8.0 Hz, 1H)	109.3	6.85 (d, *J* = 8.0 Hz, 1H)	109.3	6.85 (d, *J* = 8.0 Hz, 1H)	109.3
9	-	149.4	-	149.3	-	149.4
10	6.03 (s, 2H)	102.5	6.03 (s, 2H)	102.5	6.04 (s, 2H)	102.5
11	-	149.4	-	149.3	-	149.4
12	7.15 (d, *J* = 1.6 Hz, 1H)	106.5	7.14 (d, *J* = 2.0 Hz, 1H)	106.5	7.15 (d, *J* = 1.2 Hz, 1H)	106.5
NH	9.30 (br s, 1H)	-	8.46 (br s, 1H)	-	8.62 (br s, 1H)	-
1′	-	140.7	-	142.8	-	143.8
2′	7.74 (dd, *J* = 1.2, 8.8 Hz, 2H)	120.2	-	151.0	-	130.2
3′	7.30 (dd, *J* = 7.6, 8.8 Hz, 2H)	129.6	6.59 (d, *J* = 2.8 Hz, 1H)	99.4	6.58 (dd, *J* = 2.8 and 8.8 Hz, 1H)	108.3
4′	7.05 (m, 1H)	124.2	-	157.6	6.92 (d, *J* = 8.0 Hz, 1H)	112.0
5′	7.30 (dd, *J* = 7.6, 8.8 Hz, 2H)	129.6	6.50 (dd, *J* = 2.8 and 8.8 Hz, 1H)	104.8	-	154.8
6′	7.74 (dd, *J* = 1.2, 8.8 Hz, 2H)	120.2	8.30 (d, *J* = 8.8 Hz, 1H)	122.2	8.23 (d, *J* = 2.8 Hz, 1H)	107.9
2′-OMe	-	-	3.86 (s, 3H)	56.3	3.83 (s, 3H)	56.8
4′-OMe	-	-	3.78 (s, 3H)	55.8	-	-
5′-OMe	-	-	-	-	3.74 (s, 3H)	55.9

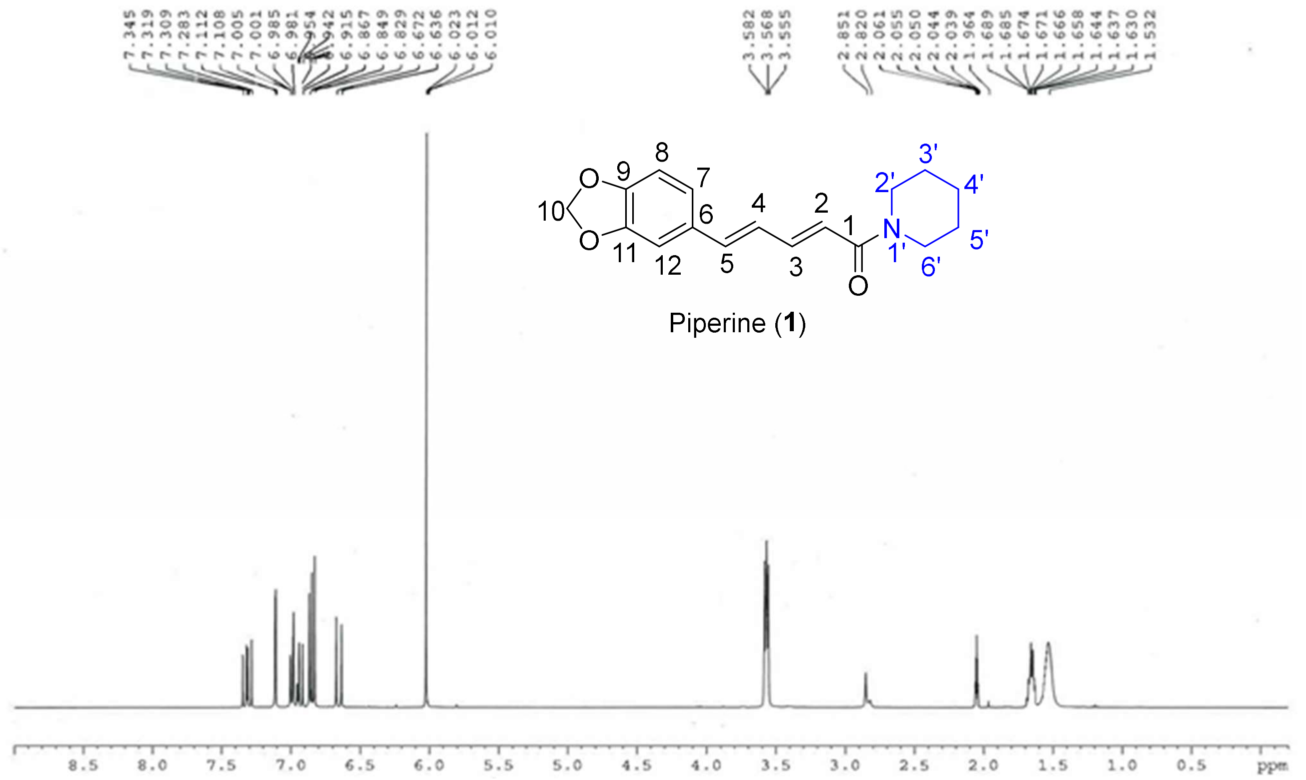

Figure S2. ^{1}H–NMR spectrum of piperine (**1**) in acetone-d_6

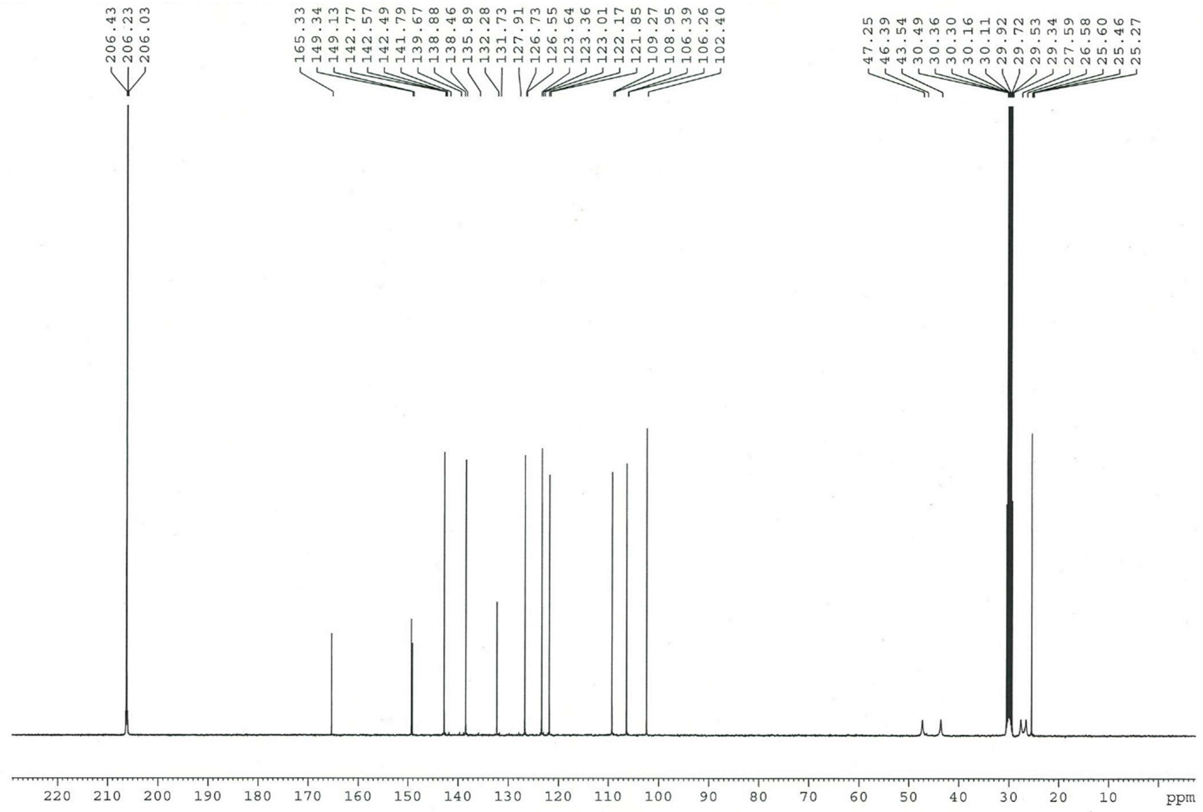

Figure S3. ^{13}C–NMR spectrum of piperine (**1**) in acetone-d_6

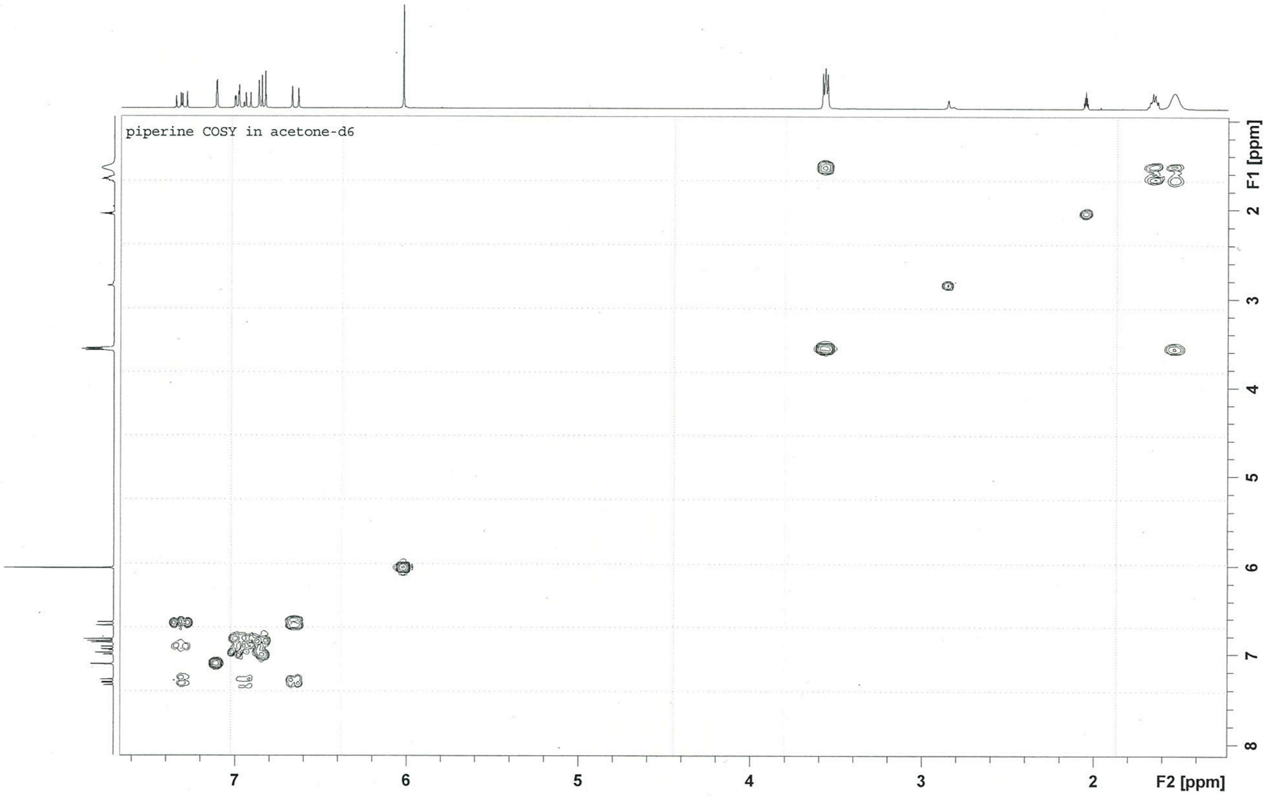

Figure S4. COSY spectrum of piperine (**1**) in acetone-*d*₆

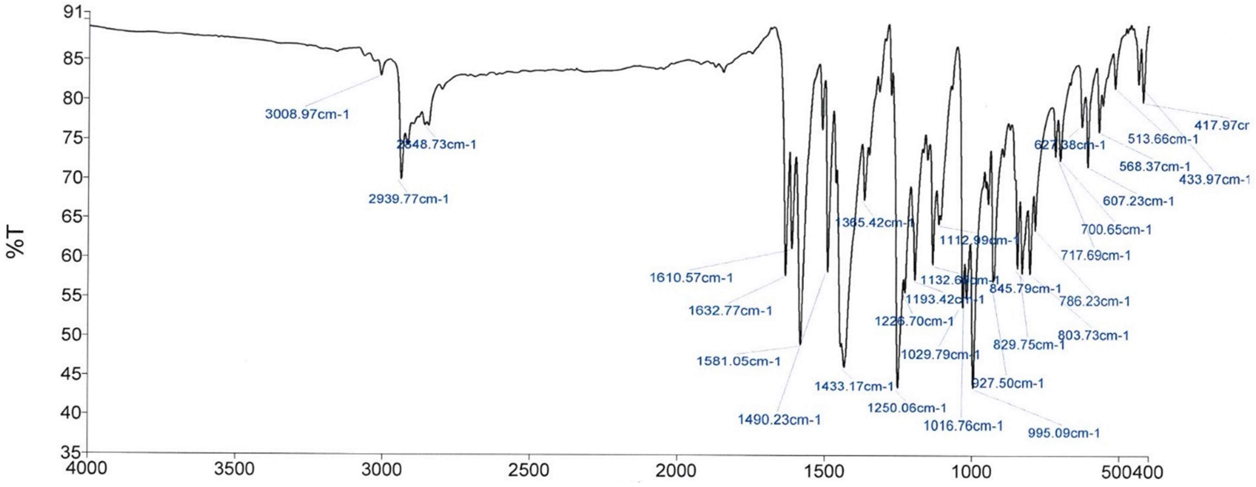

Figure S5. IR spectrum of piperine (**1**), ATR

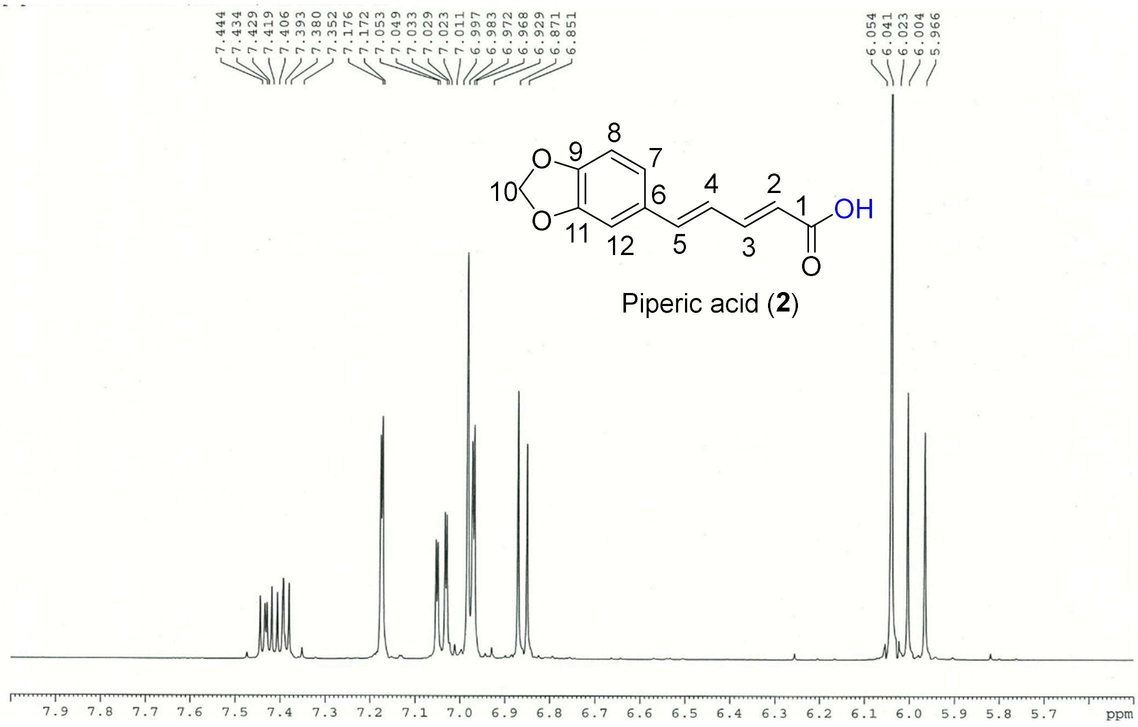

Figure S6. ^{1}H–NMR spectrum of pi peric acid (**2**) in acetone-d_6

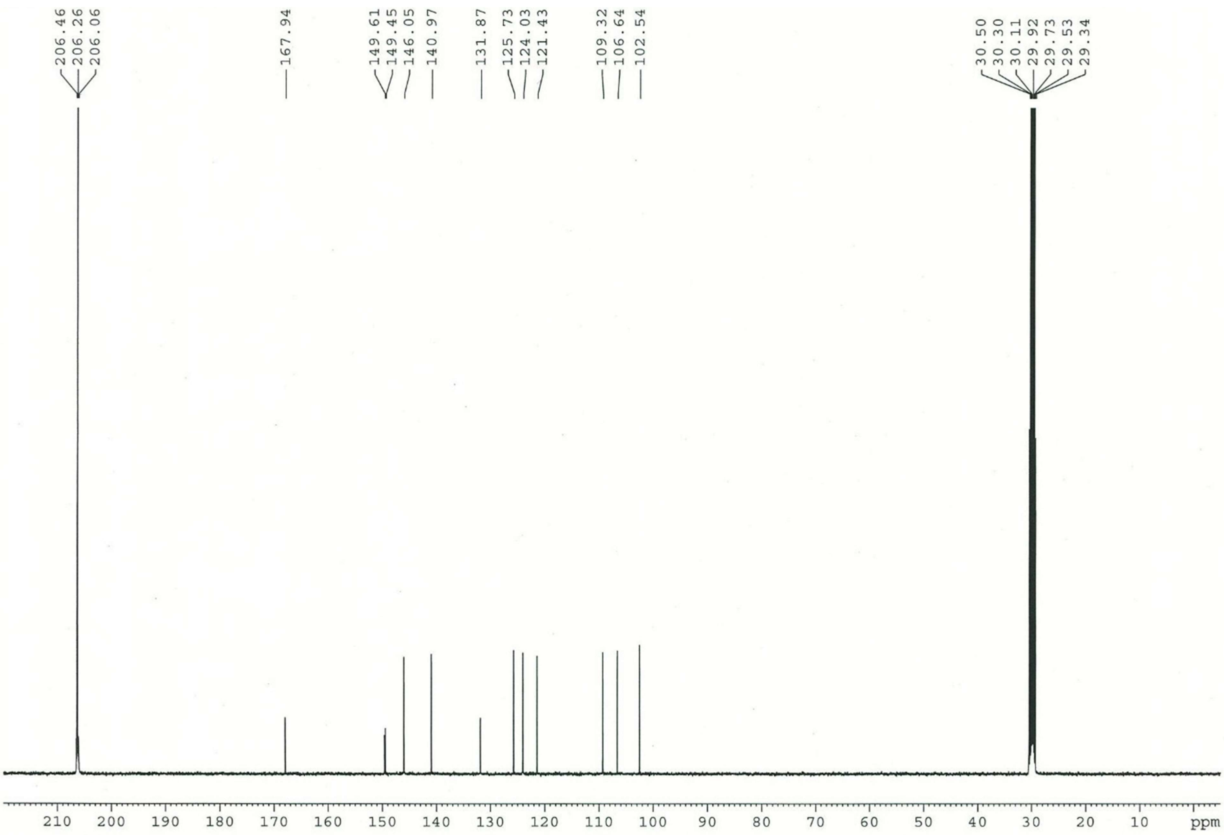

Figure S7. ^{13}C–NMR spectrum of piperic acid (**2**) in acetone-d_6

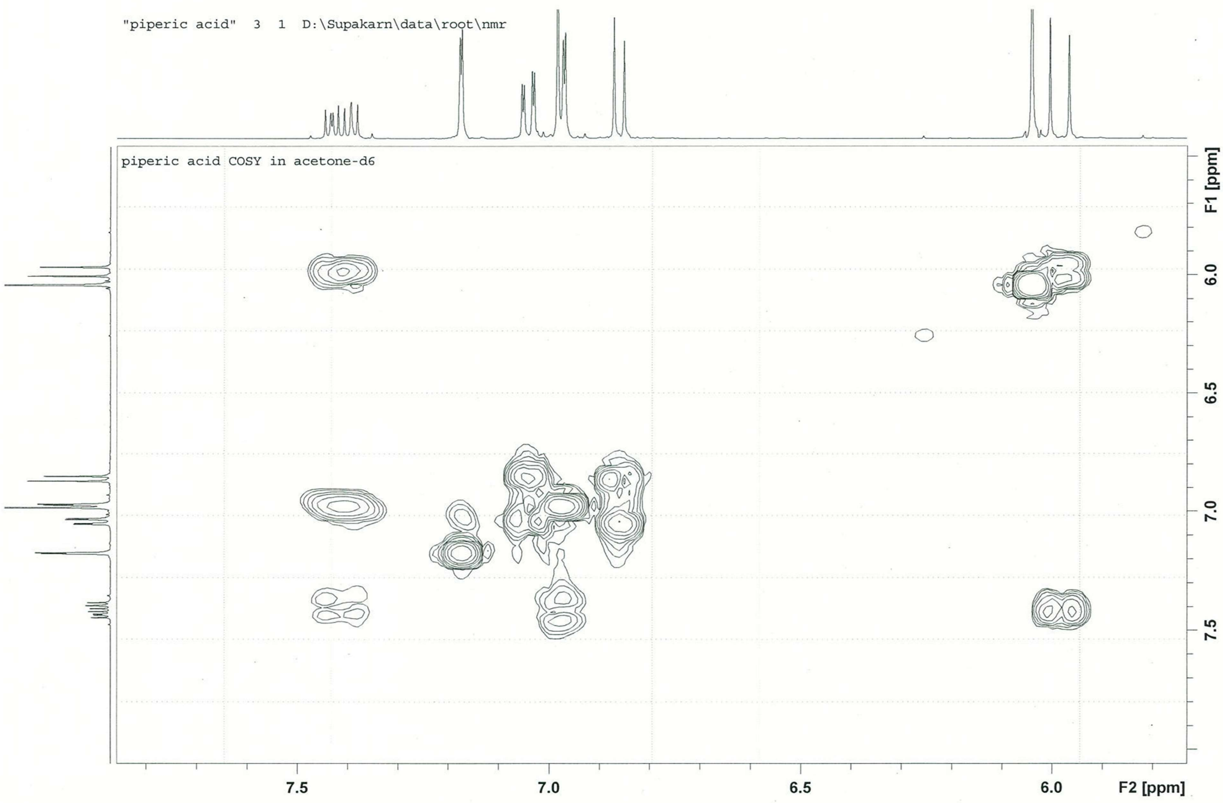

Figure S8. COSY spectrum of piperic acid (**2**) in acetone-d_6

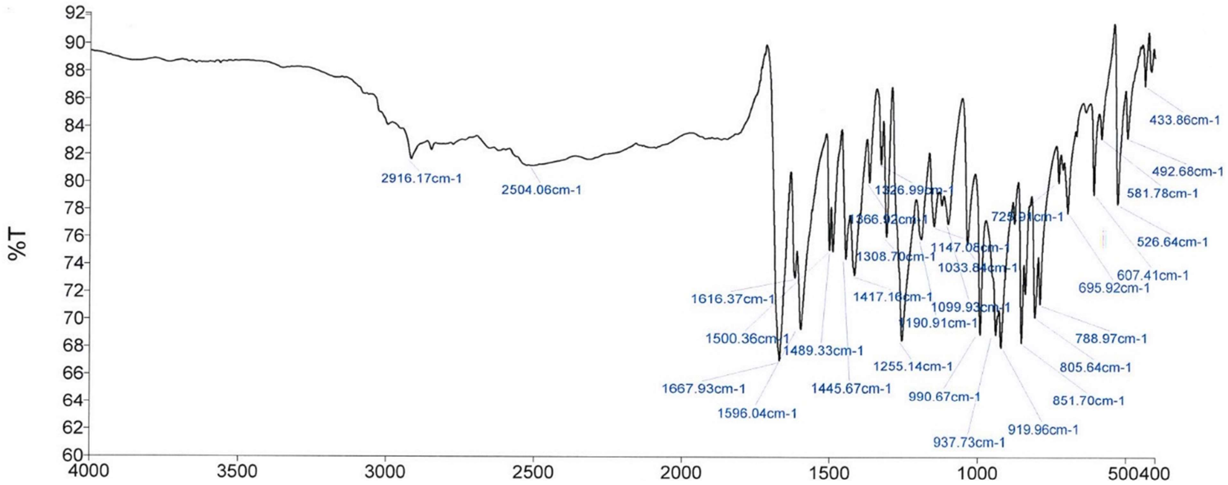

Figure S9. IR spectrum of piperic acid (**2**), ATR

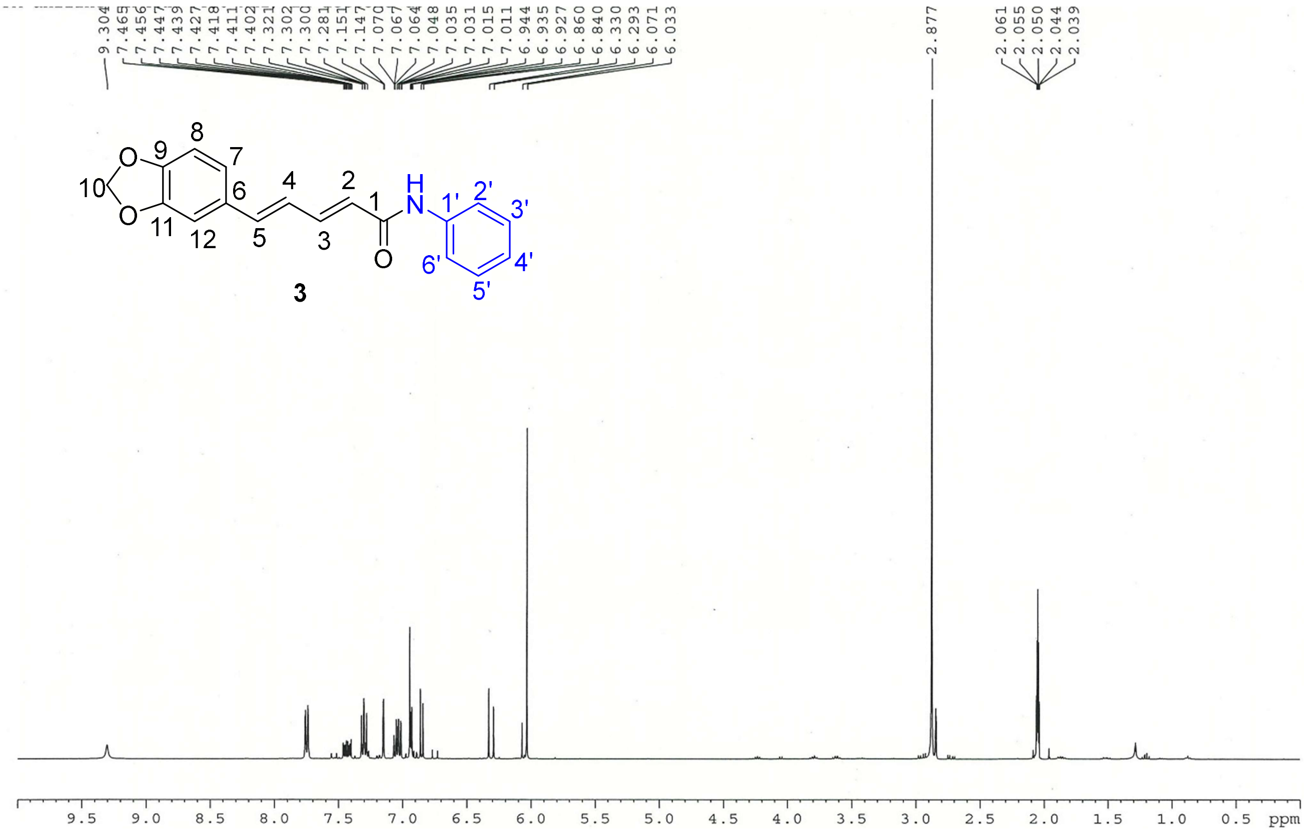

Figure S10. ^{1}H–NMR spectrum of compound **3** in acetone-d_6

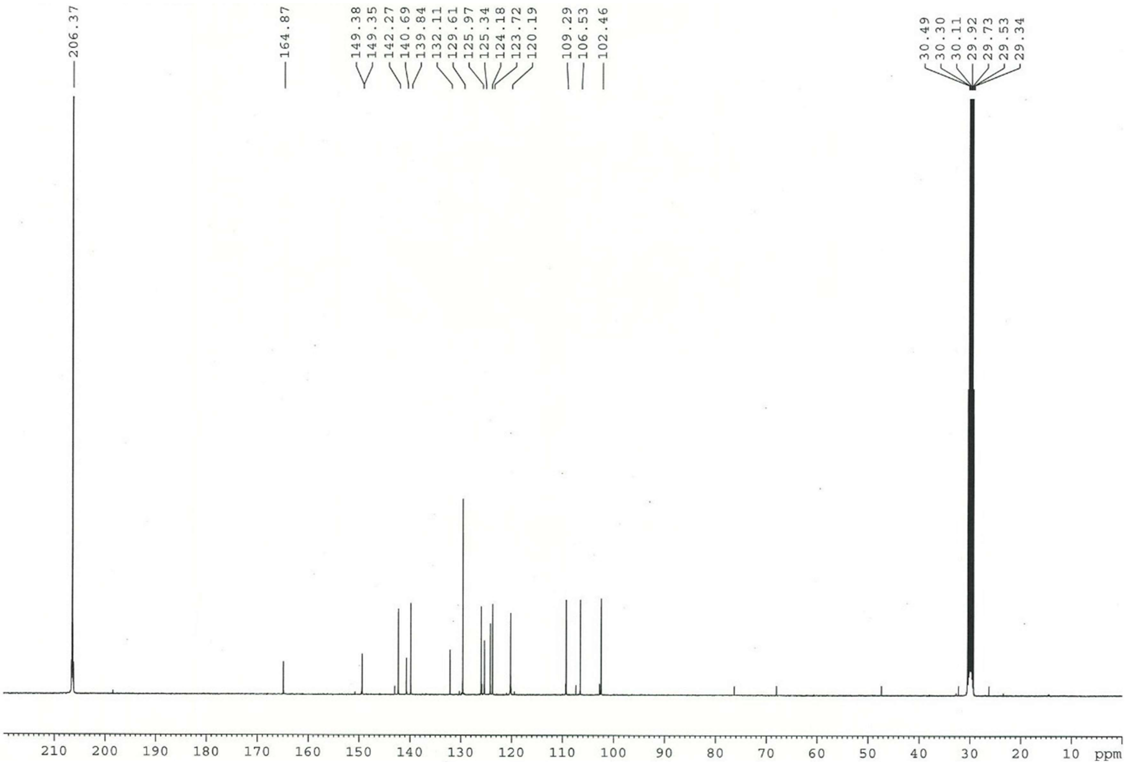

Figure S11. ^{13}C–NMR spectrum of compound **3** in acetone-d_6

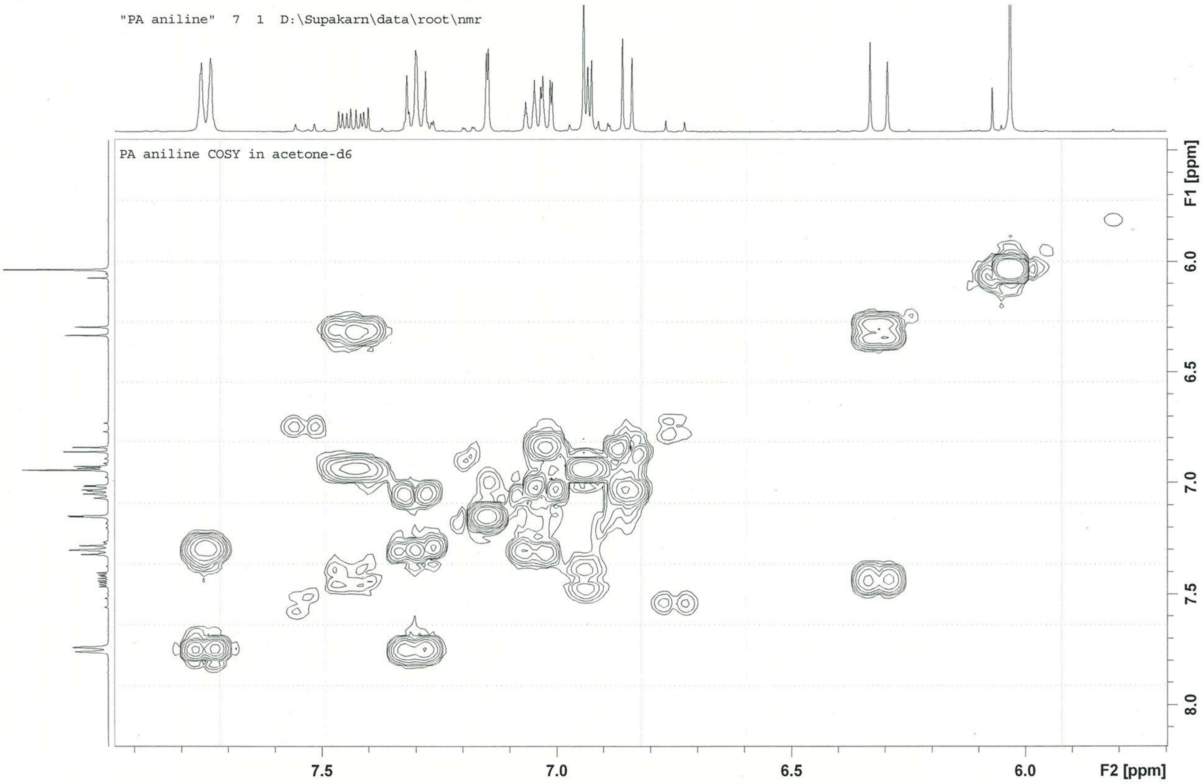

Figure S12. COSY spectrum of compound **3** in acetone-d_6

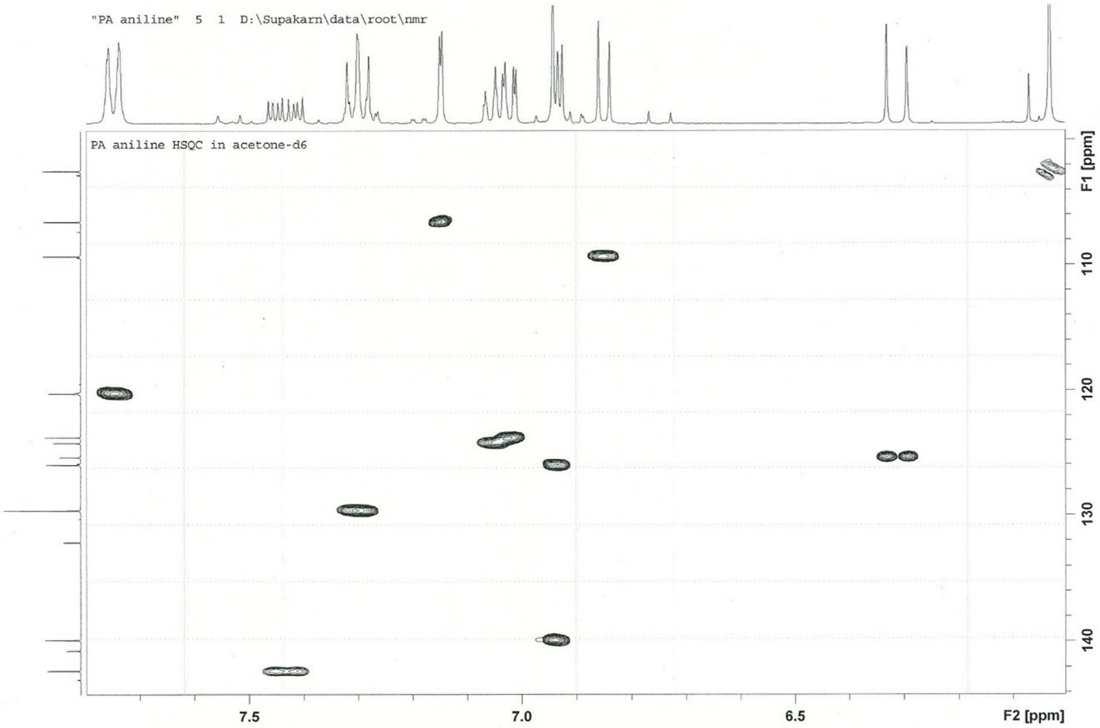

Figure S13. HSQC spectrum of compound **3** in acetone-d_6

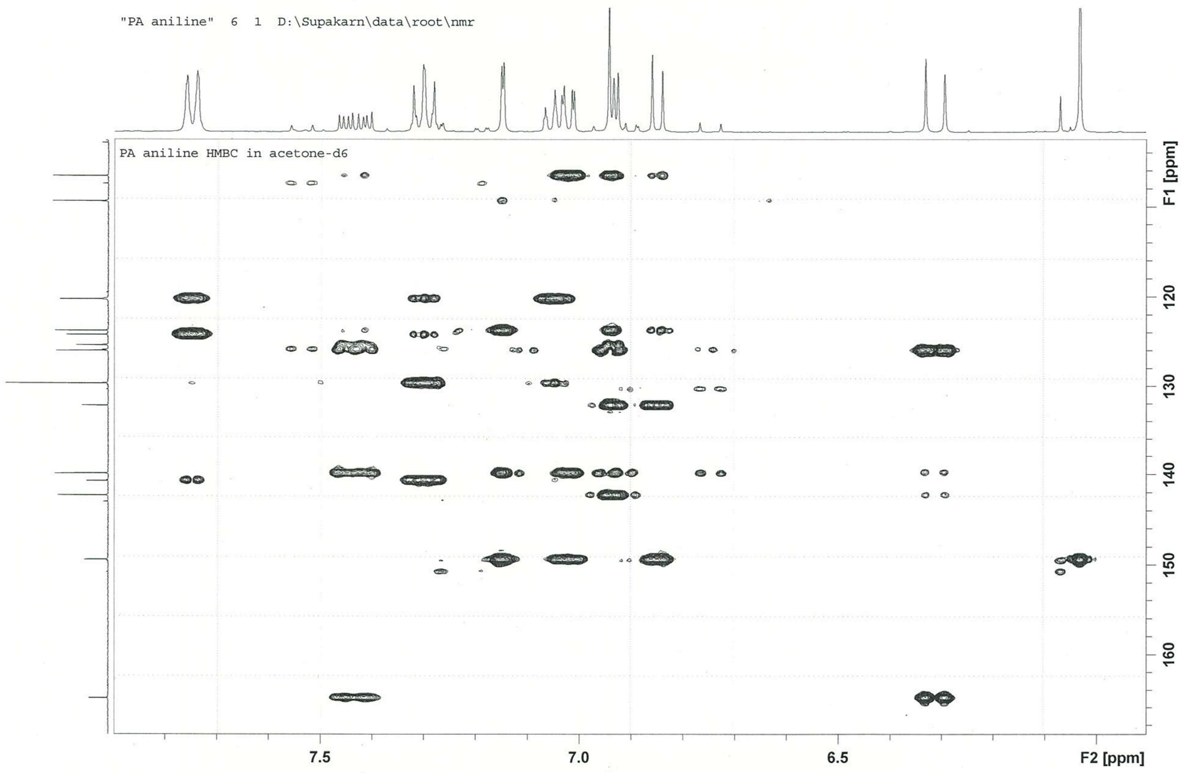

Figure S14. HMBC spectrum of compound **3** in acetone-d_6

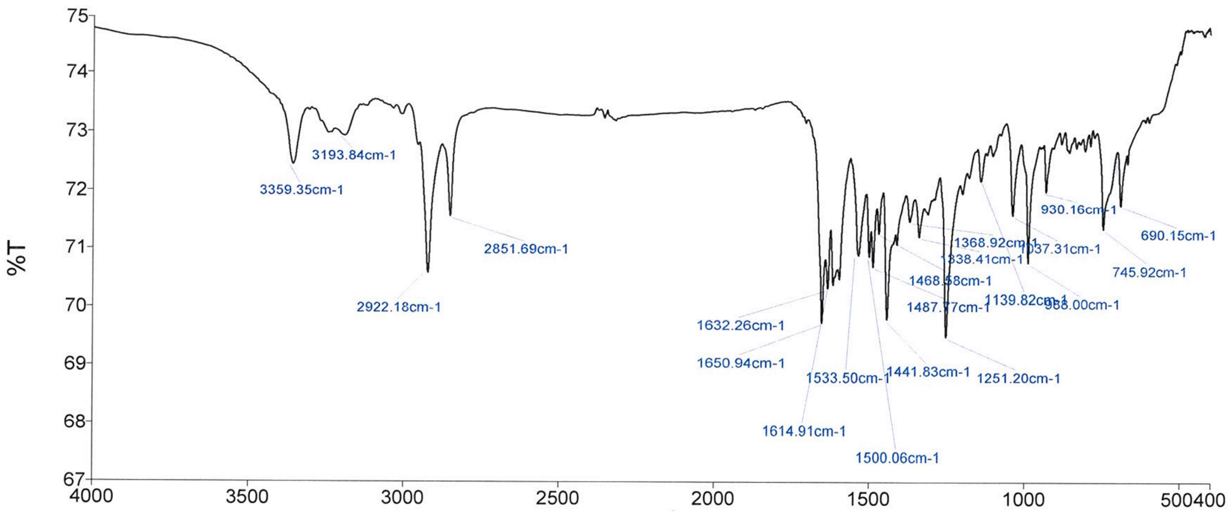

Figure S15. IR spectrum of compound **3**, ATR

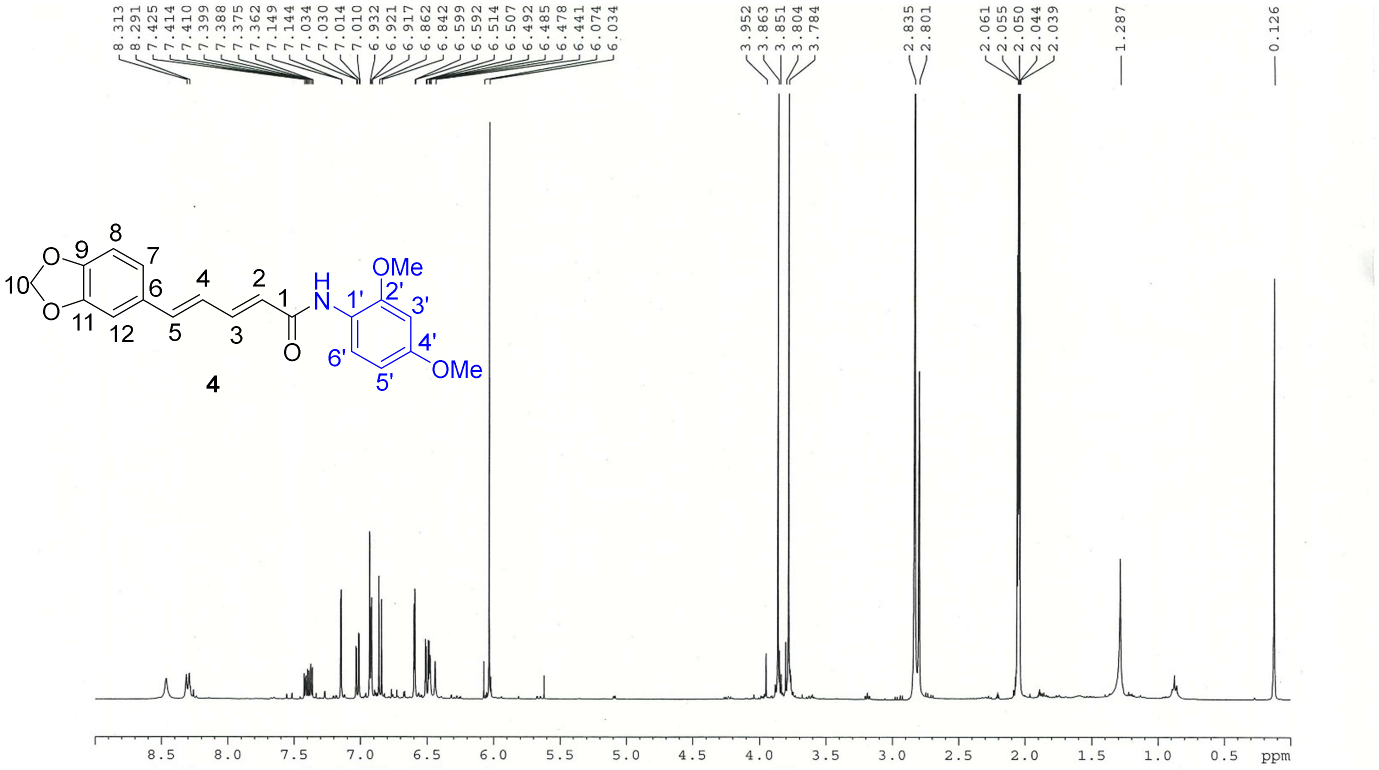

Figure S16. ^{1}H–NMR spectrum of compound **4** in acetone-*d6*

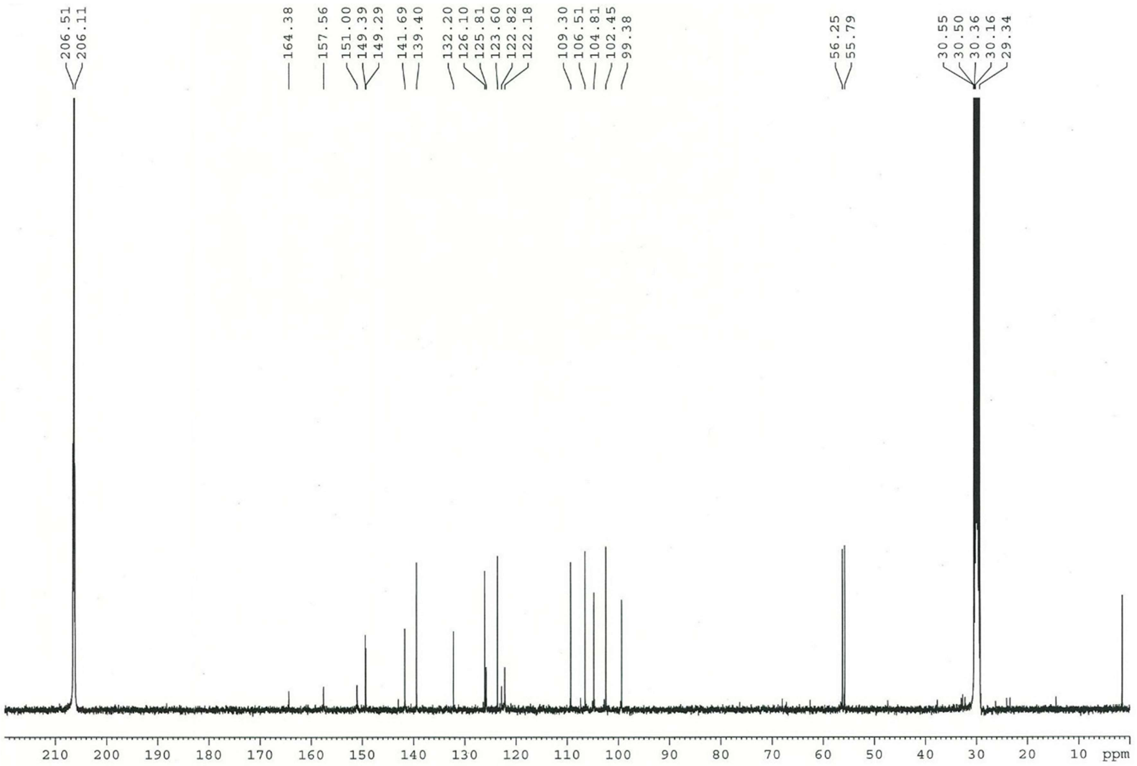

Figure S17. ^{13}C–NMR spectrum of compound **4** in acetone-*d6*

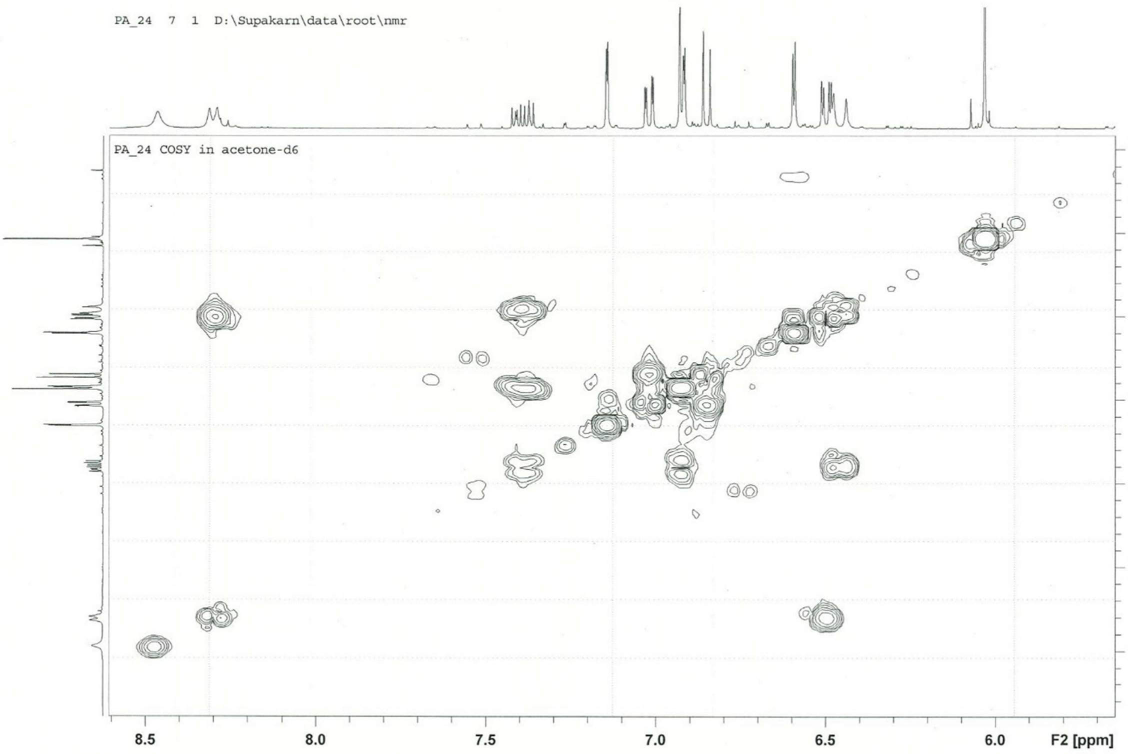

Figure S18. COSY spectrum of compound **4** in acetone-d_6

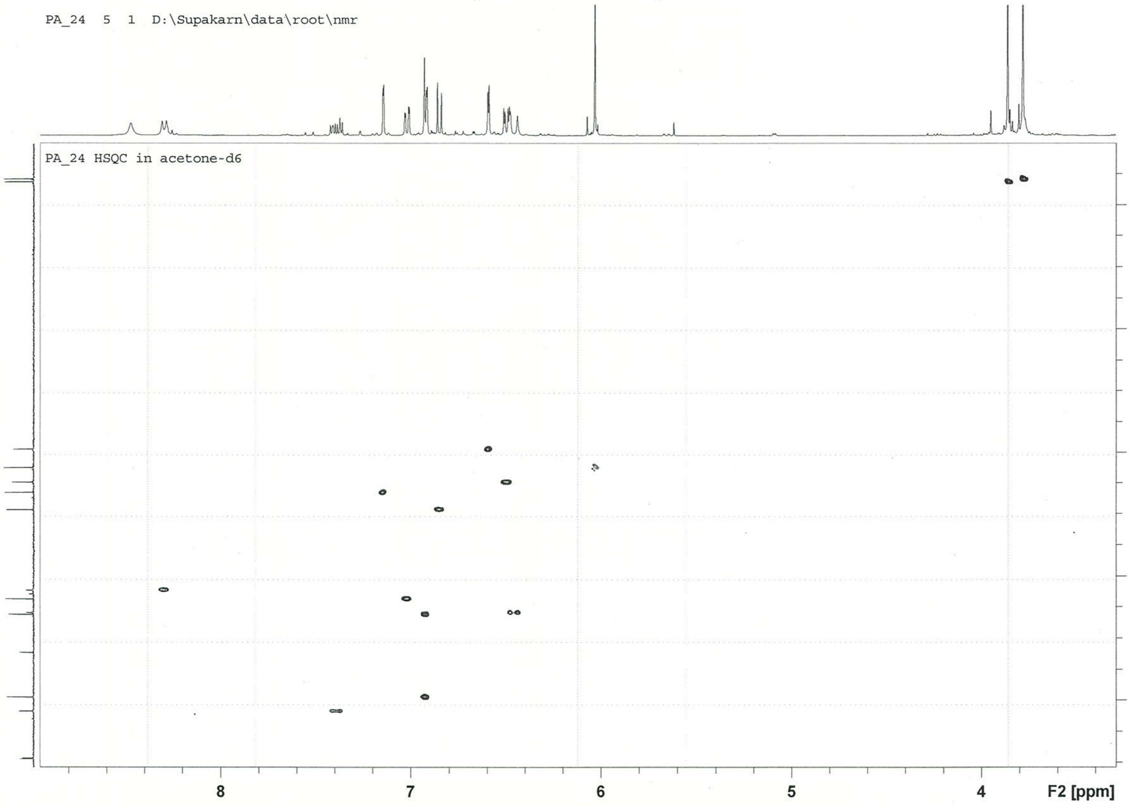

Figure S19. HSQC spectrum of compound **3** in acetone-d_6

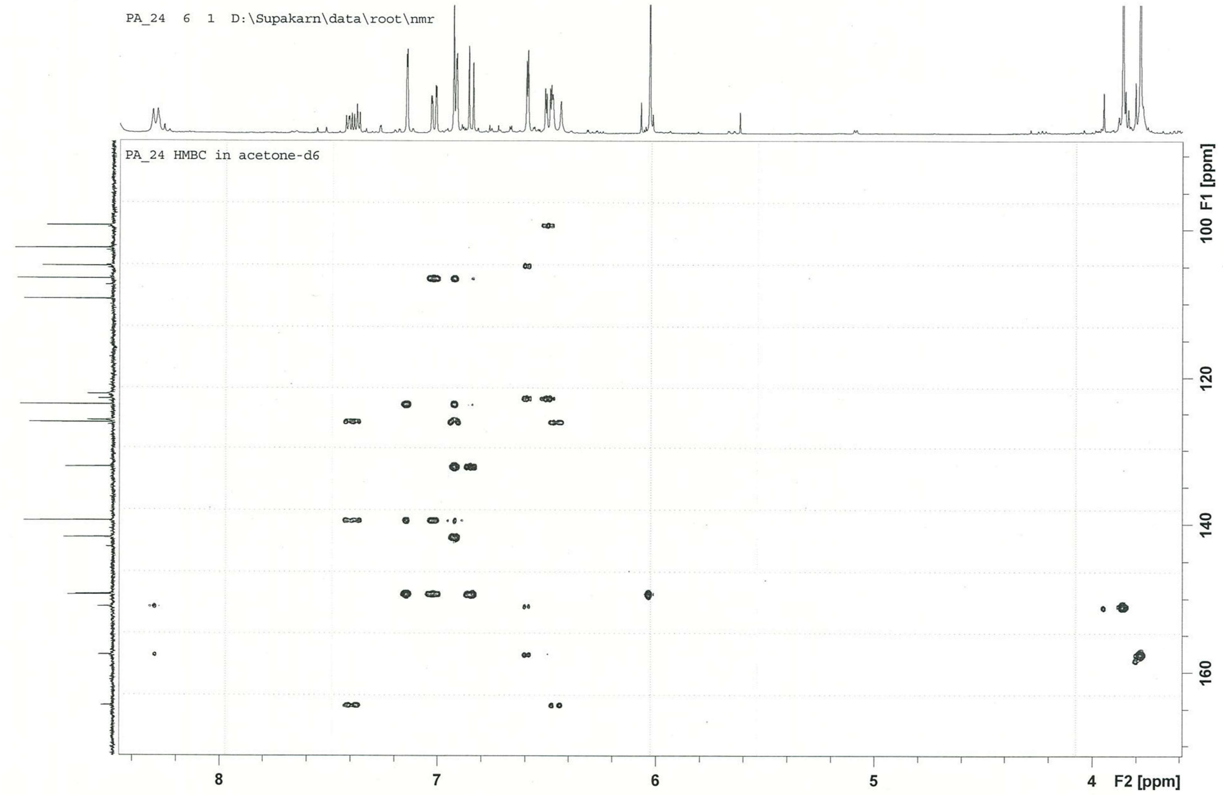

Figure S20. HMBC spectrum of compound **3** in acetone-*d6*

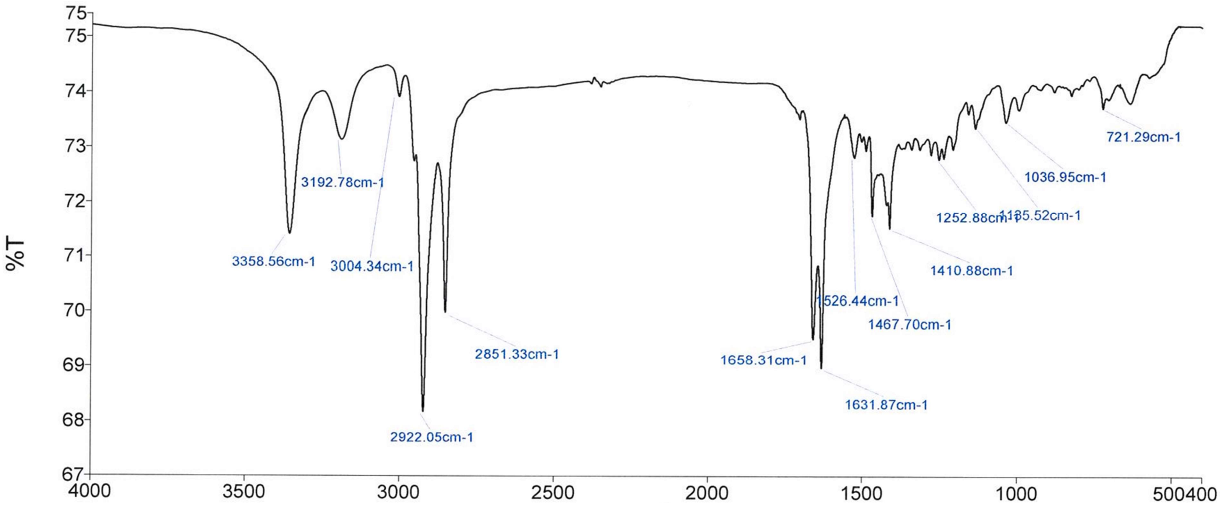

Figure S21. IR spectrum of compound **4**, ATR

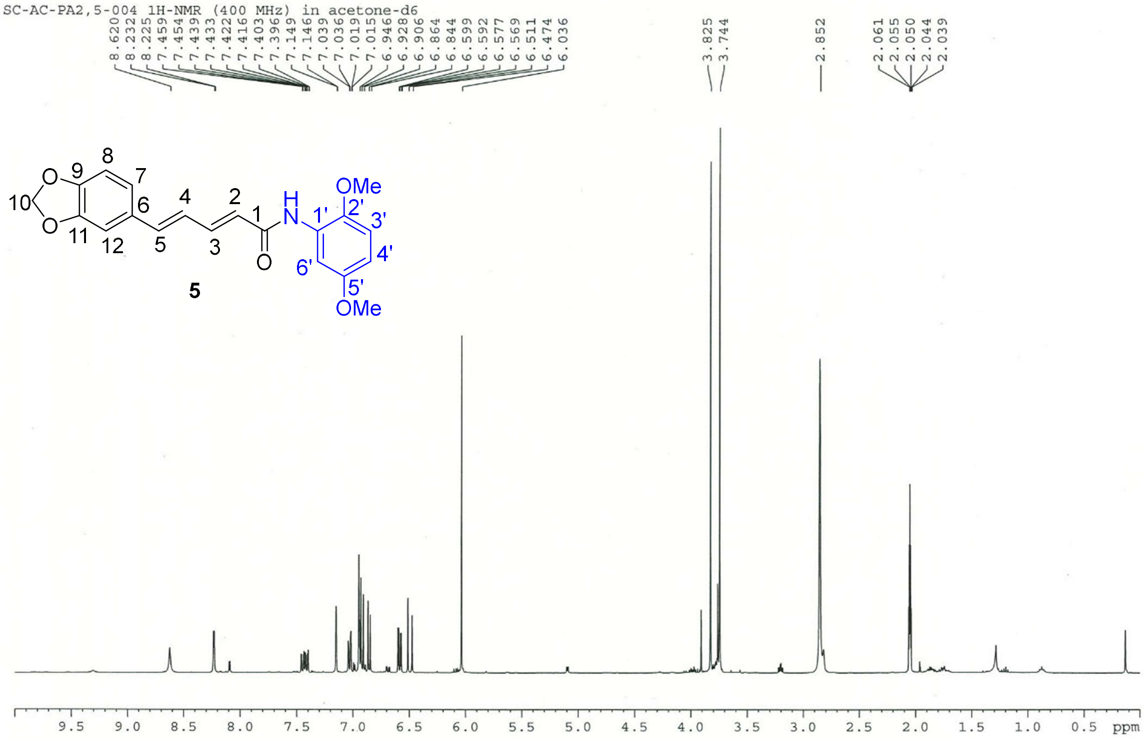

Figure S22. ^{1}H–NMR spectrum of compound **5** in acetone-d_6

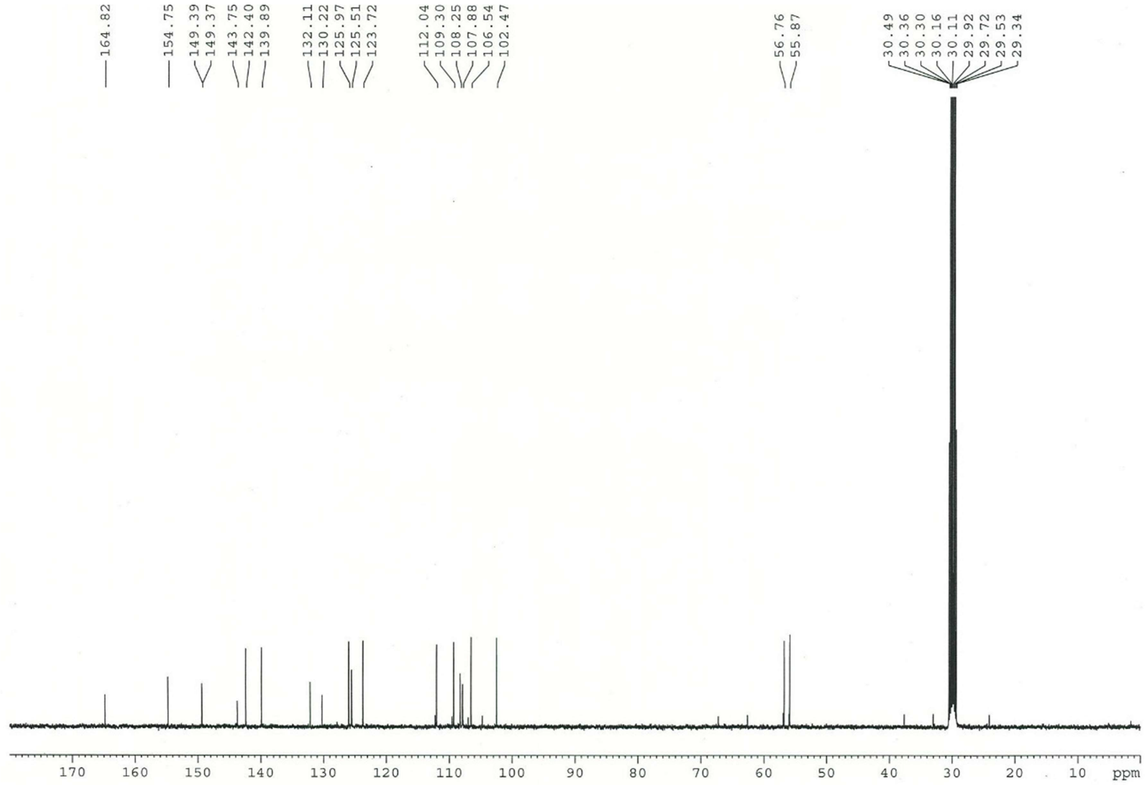

Figure S23. ^{13}C–NMR spectrum of compound **5** in acetone-d_6

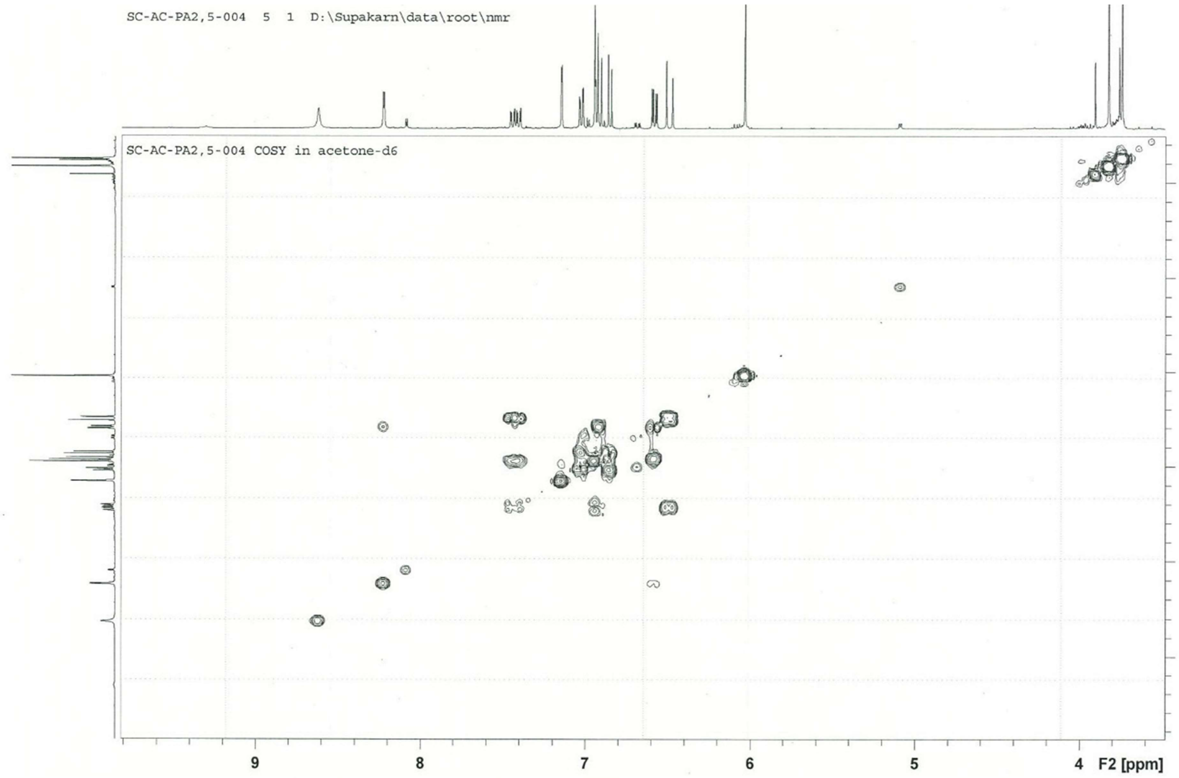

Figure S24. COSY spectrum of compound **5** in acetone-d_6

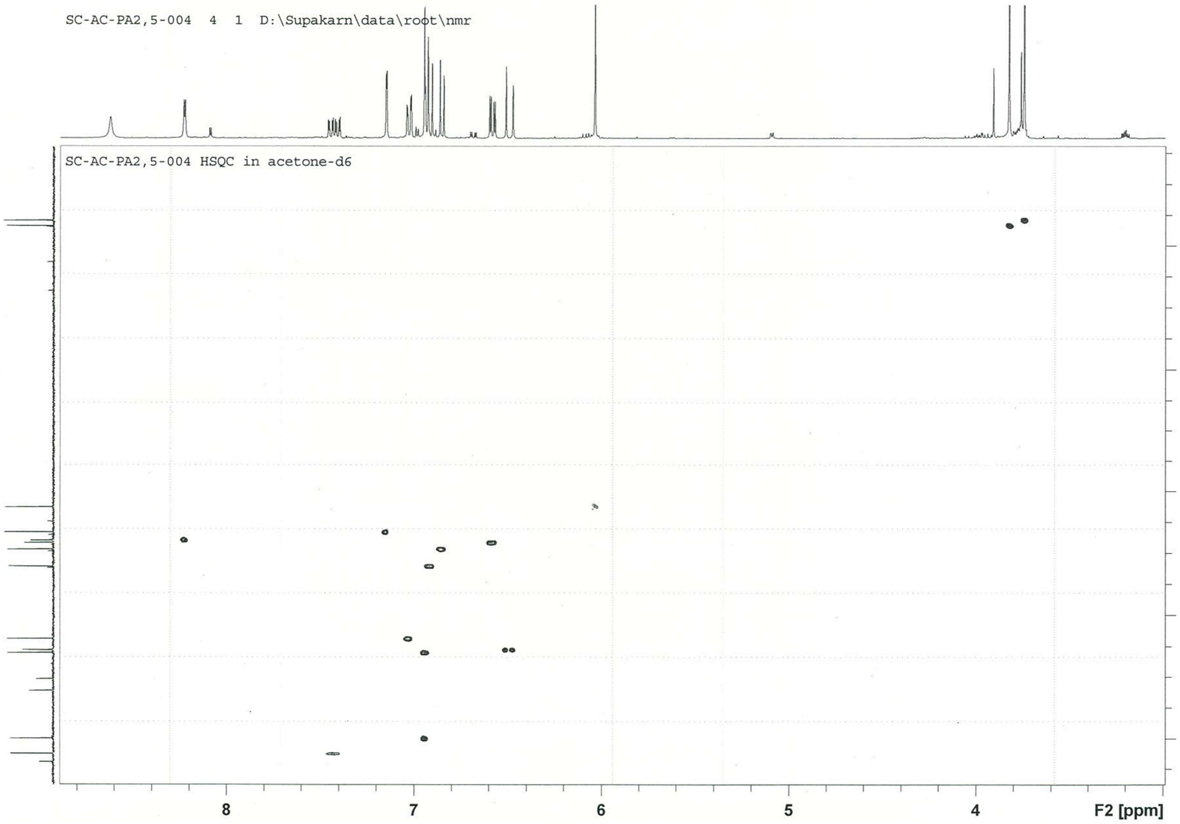

Figure S25. HSQC spectrum of compound **5** in acetone-d_6

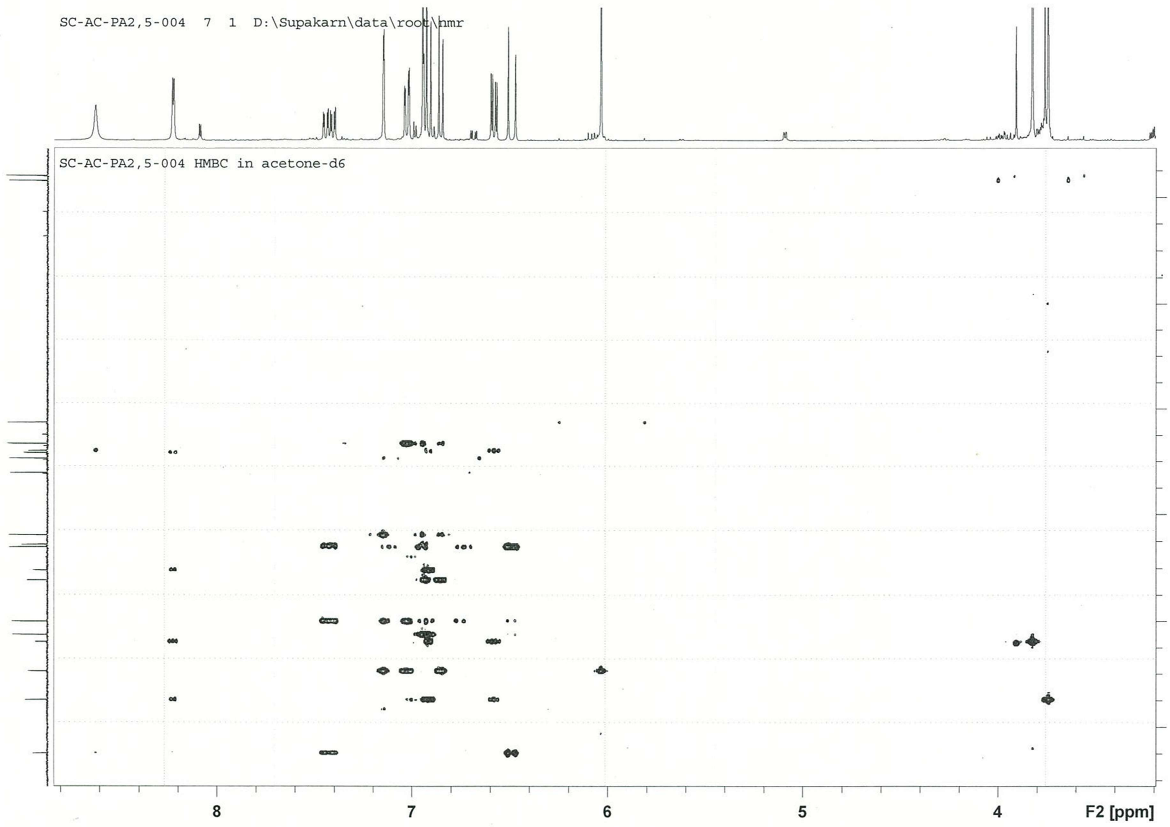

Figure S26. HMBC spectrum of compound **5** in acetone-d_6

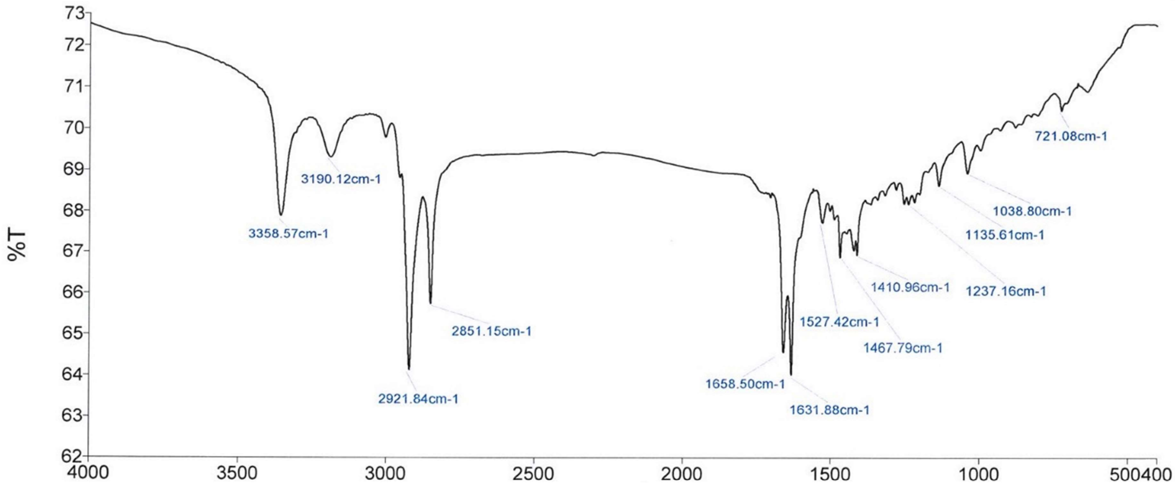

Figure S27. IR spectrum of compound **5**, ATR
